# Immunization Against Specific Fragments of Neurotrophin p75 Receptor Protects Forebrain Cholinergic Neurons in the Olfactory Bulbectomized Mice

**DOI:** 10.3233/JAD-160146

**Published:** 2016-06-22

**Authors:** Natalia Bobkova, Vasily Vorobyov, Natalia Medvinskaya, Inna Nesterova, Olga Tatarnikova, Pavel Nekrasov, Alexander Samokhin, Alexander Deev, Frank Sengpiel, Dmitry Koroev, Olga Volpina

**Affiliations:** a Institute of Cell Biophysics, Russian Academy of Sciences, Pushchino, Moscow Region, Russia; b Institute of Theoretical and Experimental Biophysics, Russian Academy of Sciences, Pushchino, Moscow Region, Russia; c School of Biosciences and Neuroscience & Mental Health Research Institute, Cardiff University, Museum Avenue, Cardiff, UK; d Shemyakin’s-Ovchinnikov’s Institute of Bioorganic Chemistry, Russian Academy of Sciences, Moscow, Russia

**Keywords:** Acetyltransferase, amyloid-beta, cholinergic mediation, Morris water maze, olfactory bulbectomy, spatial memory

## Abstract

Alzheimer’s disease (AD) is characterized by progressive cognitive impairment associated with marked cholinergic neuron loss and amyloid-β (Aβ) peptide accumulation in the brain. The cytotoxicity in AD is mediated, at least in part, by Aβ binding with the extracellular domain of the p75 neurotrophin receptor (p75NTR), localized predominantly in the membranes of acetylcholine-producing neurons in the basal forebrain. Hypothesizing that an open unstructured loop of p75NTR might be the effective site for Aβ binding, we have immunized both olfactory bulbectomized (OBX) and sham-operated (SO) mice (*n* = 82 and 49, respectively) with synthetic peptides, structurally similar to different parts of the loops, aiming to block them by specific antibodies. OBX-mice have been shown in previous studies, and confirmed in the present one, to be characterized by typical behavioral, morphological, and biochemical AD hallmarks, including cholinergic deficits in forebrain neurons. Immunization of OBX- or SO-mice with KLH conjugated fragments of p75NTR induced high titers of specific serum antibodies for each of nine chosen fragments. However, maximal protective effects on spatial memory, evaluated in a Morris water maze, and on activity of choline acetyltransferase in forebrain neurons, detected by immunoreactivity to specific antibodies, were revealed only for peptides with amino acid residue sequences of 155–164 and 167–176. We conclude that the approach based on immunological blockade of specific p75NTR sites, linked with the cytotoxicity, is a useful and effective tool for study of AD-associated mechanisms and for development of highly selective therapy of cholinergic malfunctioning in AD patients.

## INTRODUCTION

The basal forebrain cholinergic neurons, innervating the cerebral cortex and hippocampus, are involved in learning/memory functions, and their loss isassociated with impairment of cognition in Alzheimer’s disease (AD) in particular [1]. AD progression has been shown to be characterized by the attenuating activity of choline acetyltransferase (ChAT), an acetylcholine-synthesizing enzyme [2]. However, at preclinical stages of AD, ChAT activity was unaffected, which is supposed to be associated with involvement of cholinergic compensatory plasticity [3]. These findings suggest that a potential treatment for AD should be targeted at an early stage of the disease progression [4]. The most suitable model directly associated with AD *progression* is that of animals with surgically removed olfactory bulbs (OBX) that showed both typical behavioral, morphological, and biochemical AD hallmarks [5] and phasic time courses in their expression after the bulbectomy [6]. One of potential targets thought to be critically involved in the pathogenesis of AD, a neurotrophin cell surface receptor (NTR), p75NTR [7], has been shown to bind with amyloid-β (Aβ) peptides [8] that, in turn, promotes Aβ-associated neuronal dystrophy [9], and regulates both Aβ levels [10, 11], neuronal death [12], and cholinergic transmission [13]. Furthermore, in the AD brain, p75NTR expression is enhanced both in forebrain neurons and in their terminals in the cortex and hippocampus [14, 15]. Interestingly, the Aβ-induced neurotoxicity has been noted to be attenuated in these brain areas whereas the loss of cholinergic neurons in the basal forebrain to be increased [16]. The multifunctional characteristics of p75NTRs seem to be associated with the capability of Aβ-p75NTR complexes to activate a number of extra- and intracellular signaling pathways [17], implementing selectivity of Aβ-produced neurotoxicity toward cholinergic basal forebrain neurons in particular [18, 19]. Within the *intracellular* structures activated through extracellular domain of p75NTR, a death receptor (DR6) is associated with Aβ-induced apoptosis [7], and its blockade by specific antibodies (“intrabodies”) has been shown to attenuate Aβ-induced caspase 3 activation mediated by DR6 and resulting in cell death through the Jun kinase (JNK) pathway [20]. The neurotrophin binding with the extracellular domain of p75NTR may promote neuronal survival as well, either through the nuclear factor-*κ*B (NF-*κ*B) pathway or via p75NTR crosstalk with an “anti-apoptotic” tropomyosin related kinase (Trk) receptor [21]. The expression of Trk subtype NTR has been shown to be decreased in basal forebrain cholinergic neurons in AD [22]. Nevertheless, conformational transformations, revealed after neurotrophin binding with p75NTR and favorable to *simultaneous* binding of Trk and p75NTR monomers with the same neurotrophin dimer [23], might be a reasonable basis for development of a potentially effective approach to treat the AD-associated cholinergic malfunctioning. Indeed, the cysteine-rich (CR1-4) repeats of the p75NTR extracellular domain have been shown to have several binding sites for neurotrophins and Aβ [7, 24]. Therefore, we suggest that the open unstructured loops in this domain contain the sites for binding with ligands and are the most accessible targets for immunological treatment. Recently, an antibody against the extracellular domain of p75NTR has been shown to inhibit, partially, Aβ_1–42_-induced cell death in hippocampal neuronal cultures *in vitro* [18]. To unmask *in vivo* the p75NTR site(s) specifically linked with loss of ChAT-containing forebrain neurons typical for AD, we immunized OBX-mice with synthetic peptides, analogs of different p75NTR fragments of these loops. In addition, they were characterized by relatively high homology of their primary amino acid structures in mouse and human. To evaluate the efficacy of the treatment in OBX-mice, the extents of both spatial memory improvement in a Morris water maze and recovery of ChAT-positive cell density in the basal forebrain were analyzed. Recently, this immunological approach has allowed the revealing of the Aβ-binding sites on *α*7-nicotinic acetylcholine and prion receptors whose blockade with specific antibodies suppressed the progression of AD-linked neurodegeneration in OBX mice [25, 26].

In our present study with nine selected amino acid fragments of p75NTR, only immunization against two of sequences 155–164 and 167–176 effectively protected cholinergic mediation in the forebrain and, as a result, cognitive abilities of OBX-mice fromAD-like neurodegeneration.

## MATERIAL AND METHODS

One hundred and thirty one male mice (NMRI, Charles River, USA) bred in a colony under controlled barrier conditions were used in this study. The animals were given food and water *ad libitum* and reared in a standard 12-h/12-h day/night cycle. The procedures were carried out in accord with the principles enunciated in the Guide for Care and Use of Laboratory Animals, NIH publication No 85-23, and approved by the Institutional Ethical ReviewCommittee.

### Olfactory bulbectomy (OBX)

Nearly 3-month-old mice (25–30 g) were anaesthetized with Nembutal (40 mg/kg, i.p.) and a hole of 2 mm in diameter was drilled medially over the olfactory bulbs (2.0 mm anterior to bregma). The bulbs were aspirated carefully under visual control through a blunt needle attached to a water pump. Sham-operated mice were treated similarly with exception of the bulbectomy.

### Experimental protocol

The first immunization procedure (Day 1) initiating production of antibodies against one of nine selected p75NTR fragments (32–46, 39–47, 66–72, 86–93, 97–105, 115–122, 147–154, 155–164, and 167–176) was performed on 2-month old mice, which were then bulbectomized or sham-operated on Day 25 and immunized repeatedly on Day 43. For the immunization, emulsions of PBS solution of a KLH-peptide conjugate (Sigma-Aldrich, USA) mixed with either CFA or IFA (the 1st or the 2nd immunization, respectively; Disco laboratories, USA) were subcutaneously (s.c.) injected at a dose of 100 μg of the protein per mouse in the proximal part of its tail (in control groups, vehicle was used). Starting on Day 53 mice were trained in a Morris water maze: Initially, in 3 trials, to reach a *visible* saving platform and then, in 4 daily trials for 5 days, with an *invisible* (submerged) platform. Finally, on Day 58, a memory test followed by the brain preparation for further histological and histoimmunochemical analysis was performed. The serum samples for the antibody titer estimation were taken from the eye vein on Day 52 and kept at –20°C for further analysis (see results in Table 1). As controls for the p75NTR fragments, KLH (Keyhole Limpet Hemocyanin) conjugate of ***SQDRHKQKIIAPAKQLL*** peptide corresponding to the fragment of 197–213 of VP1 protein from foot-and-mouth disease virus (VP1), and KLH along were used. The Maxisorp’s 96-wells plates (Nunc, Denmark) were coated with 20 μg/ml ovalbumin-conjugated peptide in 0.05 M sodium bicarbonate buffer (pH 9.5); blood serum samples in successive twofold dilutions were added. Then the incubation with anti-mouse immunoglobulin antibodies conjugated to horseradish peroxidase was carried out. The substrate solution (0.05% hydrogen peroxide and 0.05% o-phenylenediamine in citrate buffer, pH 5.0) was added to the wells for visualization. The reaction was stopped by adding 10% sulfuric acid, and optical absorption was measured at 492 nm. The highest dilution of the sera with OD >0.1, which was still positive and exceeded the level of preimmune serum more than twofold, was taken as the antibody titer.

### Spatial discrimination testing (Morris water maze)

On the 28th day after surgery (Day 53 after 1-st immunization), the learning/memory abilities of the mice were tested with the Morris water maze in a circular water pool (90 cm in diameter, 50 cm in height), filled to a depth of 40 cm with water (33°C ± 1) rendered opaque by the addition of powdered milk. Four equal virtual sectors in the pool were arbitrary indicated as I, II, III, and IV. Each mouse was first tested on its capability to reach a *visible* platform (0.5 cm *above* the water surface, 8 cm in diameter). All mice used in this study demonstrated similar swimming abilities in reaching the visible platform. A day later and for 5 days, each mouse was trained (4 trials per day, with an intertrial interval of 25–30 min) to find the platform centered in sector III but submerged 0.5 cm *below* the surface. The animals were placed in a randomly chosen sector and allowed a maximum of one minute to find the platform followed by a 15-s rest period, once on the platform, before being dried and relocated into the home cage. An unsuccessful mouse was gently redirected to the platform after exceeding the 60-s limit. On the day after the end of the training trials, a 60-s memory test, with the platform temporarily removed from the pool, was given to assess spatial memory in mice for the platform’s former location (in the sector III). The total time spent in the target sector III during the test were computed using a home-developed tracking system by using of a digital camera (Logitech QuickCam 3000, 800×600 pixels, 15 fps) located 90 cm above the pool.

### Histology/morphology and choline acetyltransferase (ChAT) evaluation

Immediately after memory test session, all mice were deeply anaesthetized with overdose of Nembutal (60 mg/kg, i.p., Sigma, USA), their brains were perfused through the left cardiac ventricle with a phosphate-buffered solution and verified on the extent of the bulbectomy. Only the brains with both bulbs fully removed (>90% of their estimated volumes) and no damage to prefrontal cortices were accepted for further histological determination of a population ratio of pathological versus normal neurons and immunohistochemical analysis of the basal forebrain ChAT-containing cells. The animals with insufficient bulbectomy were excluded from further analysis of results of their learning/memoryabilities.

The brain blocks containing the cortex and hippocampus, preliminarily fixed in 4% paraformaldehyde (dissolved in 0.1 M PBS, pH 7.4) for 24 h at RT, were used for morphological and immunohistochemical evaluation. Each block was cut on a freezing microtome (Reicher, Austria) into 10-μm and 30-μm coronal sections collected at 70-μm intervals. The thin (10-μm) slices were stained with 0.1% Cresyl Violet, and their magnified (at 20x or 40x, optical microscope Nikon Eclipse E200) and digitized (DXM1200 camera) images were used for further morphological and morphometric examinations. All neurons were divided into a normal category and several pathologies characterized by cytolysis, karyolysis, pyknosis, and vacuolization. The neurons from different categories were counted in each investigated brain areas as a percentage of total cell population observed in 10–12 randomly selected fields (100 cells in each fields) in 3-4 sections per mouse.

The thick (30-μm) slices were used for the analysis of the ChAT-containing cells in the *nucleus basalis magnocellularis* (NBM) [27], the equivalent of the primate *n. basalis* of Meynert [28]. The slices were incubated in PBS with consecutive addition of a) 1% H_2_O_2_, for 30 min; b) 5% bowing serum albumin, for 2 h; c) polyclonal rabbit antibodies against ChAT (ab68799, Abcam, Cambridge, USA) with 1:200 dilution, for 12 h at 4°C; d) biotinylated coat antibodies against rabbit’s *γ*-globulines with 1:100 dilution, for 2 h at 20°C; e) avidine-biotine complex with 1:100 dilution, for 1 h at 20°C. The avidine-biotine complex’s peroxidase activity was revealed by incubation of slices with both 0.5% 3,3’- diamino-benzidin-tetra-hydrochloride and 0.02% H_2_O_2_ in PBS at 20°C. Finally, these slices were putted into the Apati’s media followed by their examination at 40-x magnification on an optical microscope Nikon Eclipse E200 with digital DXM1200 camera. The images of slices with NMB were stored for further quantification of ChAT-immunopositive neurons. To evaluate the extent of the bulbectomy-produced damage in NBM a neuronal apoptosis was analyzed by use of two sorts of staining: Conventional Nissl, for the whole cells, and DAPI, demonstrating nuclear chromatin destruction that is directly associated with apoptosis. In DADL analysis, only roundish intracellular nuclei with size of >10 μm, typical for neurons, were used. The number of apoptotic neurons versus total number of cells in 3-4 brain slices with NBM was estimated and presented in percentage for each of the p75NTR fragments used.

### Peptide synthesis

Peptides were synthesized using reagents and amino acid derivatives produced by Merck (Germany) and Fluka (Switzerland). Immunological studies were carried out with the keyhole limpet hemocyanin (KLH) solution in PBS at a concentration of 5 mg/ml (Sigma–Aldrich, USA), 25% aqueous solution of glutaric dialdehyde (Sigma, USA), Freund’s complete adjuvant, Freund’s incomplete adjuvant (Difco Laboratories, USA), goat antimouse immunoglobulin antibodies conjugated to horseradish peroxidase (Imtek, Russia), and 96-well Maxisorp plates (Nunc, Denmark). Peptides were chosen according to the sequence of the human p75 (TNR16_HUMAN) UniProtKB), and their synthesis was carried out on Wang resin, using the Fmoc/But-scheme. All peptides contained additional C-terminal glycine residue. TBTU/ DIEA method was applied for elongation of peptide chain. Cleavage of peptides from the resin was carried out in a mixture of TFA (94%), triisopropylsilane (1%), 1,2-ethane dithiol (2.5%), and water (2.5%) for 2 h. The peptides were purified by reverse phase HPLC in Phenomenex Jupiter 10 μ C18 300A 250×10 mm in an acetonitrile gradient from 10 to 70% in 0.1% TFA solution. Synthetic peptides were characterized by the data of amino acid analysis, analytical HPLC, and mass spectrometry.

### Peptide conjugates with KLH and ovalbumin

The KLH-conjugated peptides were prepared as follows: 1 mg of the peptides was added to 1 ml of KLH solution in PBS (5 mg/ml, pH 7.4) and stirred for 30 min. Then 50 μl of 0.25% glutaraldehyde solution was added by drops, incubated for 16 h, and dialyzed against PBS for 18 h, with the buffer changed three times. The ovalbumin conjugate was prepared by dissolving 1 mg of the peptide in 500 μl of PBS, with the addition of 2 mg of ovalbumin. Then 50 μl of hydrochloride N-(3-dimethylaminopropyl)-N’-ethylcarbodiimide solution (0.08 mg/ml) was added by drops during 30 min, incubated under stirring (16 h) and dialyzed against PBS with the buffer changed two times.

### Statistics

Differences in behavioral, immunohistochemical, and morphometric parameters were evaluated by either non-parametric Mann-Whitney U-test, one/two-way ANOVA for repeated measures or two-tailed Student’s *t*-test, when appropriate (*p* < 0.05 was deemed statistically significant). The data in the text and figures are expressed as mean ± SEM.

## RESULTS

### Selective effects of immunization against different p75NTR fragments on acetylcholine-synthesized capability of neurons in the nucleus basalis magnocellularis (n. Meynert)

**Fig.1:**
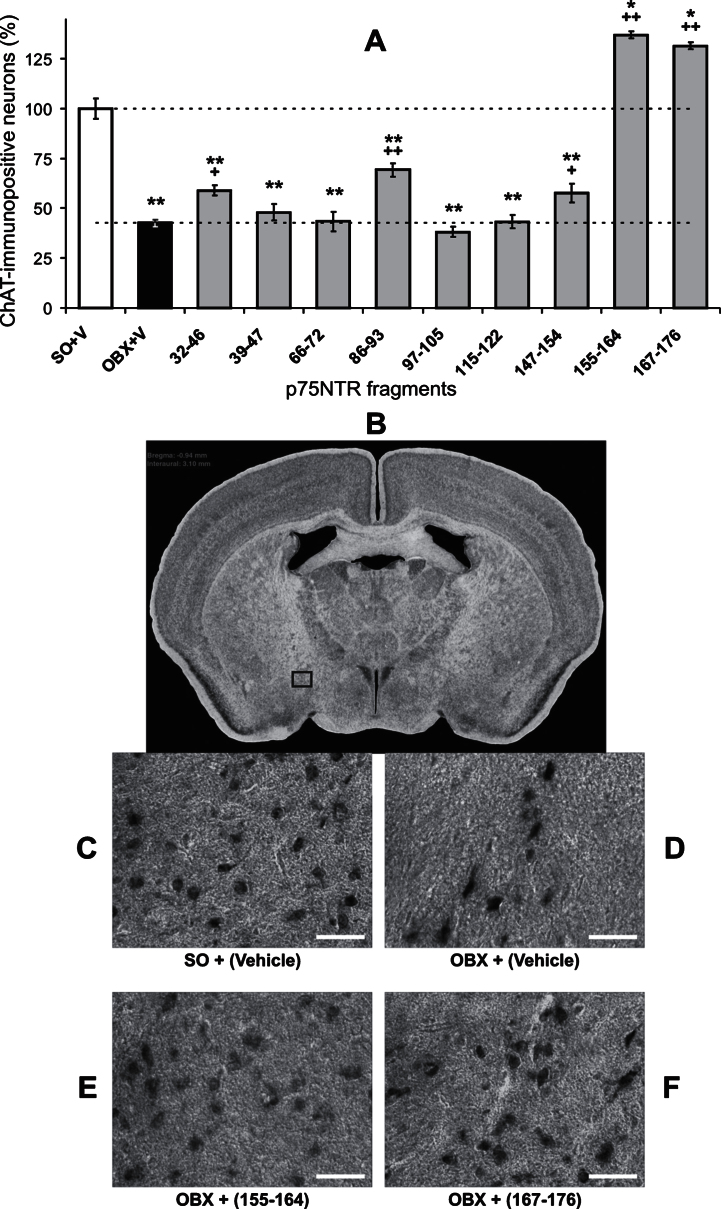
Detrimental effect of olfactory bulbectomy (OBX) in mice on the density of choline acetyltransferase (ChAT)-containing neurons in the forebrain, beneficial effects of the immunization against the p75NTR amino acid residues of 32–46, 86–93, and 147–154, and overgrowing effects of the treatment with 155–164 and 167–176 fragments. The immunoreactive labeling against ChAT in the *nucleus basalis magnocellularis* on the representative examples (taken from the area marked by a rectangle on B) demonstrates decreased number of the labeled cells after the bulbectomy (c.f., C and D) and their recovery after the immunization against the fragments of 155–164 (E) and 167–176 (F). White horizontal bars in C-F are the scale bars of 50 μm. On A, error bars show 1 SEM; star or plus denote significant differences versus either SO or OBX, respectively (one and two markers are *p* < 0.05 and 0.01, Mann-Whitney U-test). The brain slice on B was modified from [40].

To test the idea of possible brain protection against amyloid-produced toxicity by use of an immunological blockade of Aβ binding with the neurotrophin receptors, we have studied forebrain cholinergic neuron functioning in the olfactory-bulbectomized (OBX) mice, which have been shown to be characterized by enhanced Aβ levels in the brain [29]. In this section, we analyzed 498 brain slices from 48 mice distributed within the groups as follows (slices/mice): SO (61/7), OBX (86/6) or OBX after the immunization with synthetic peptides, the analogs of the p75NTR fragments. They were the following (slices/mice): 32–46 (94/6), 39–47 (25/3), 66–72 (24/3), 86–93 (46/4), 97–105 (44/4), 115–122 (27/3), 147–154 (35/4), 155–164 (24/4), and 167–176 (32/4). In the immunohistochemical staining of the brain slices by use of specific antibodies against choline acetyltransferase (ChAT), a key enzyme for acetylcholine synthesis, significant depletion of the neuronal population of the forebrain ChAT-positive neurons in NBM was revealed in OBX- versus SO-mice one month after the bulbectomy (Fig. 1A). After the immunization with synthetic analogs of the p75NTR fragments, only four of them with residuals of 32–46, 86–93, 155–164, and 167–176 were statistically effective in protecting the cholinergic cells from their depletion observed in non-immunized OBX-mice, with maximal effects associated with peptides of 155–164 and 167–176 (Fig. 1A). This is clearly visible on individual slices with enhanced densities of ChAT-containing neurons in NBM in two OBX-mice, immunized against 155–164 and 167–176 fragments of p75NTR (c.f., Fig. 1E and F versus D, respectively) and confirmed by one-way ANOVA (F_10,487_ = 74.6, *p* < 0.001) for the whole set of slices.

**Fig.2:**
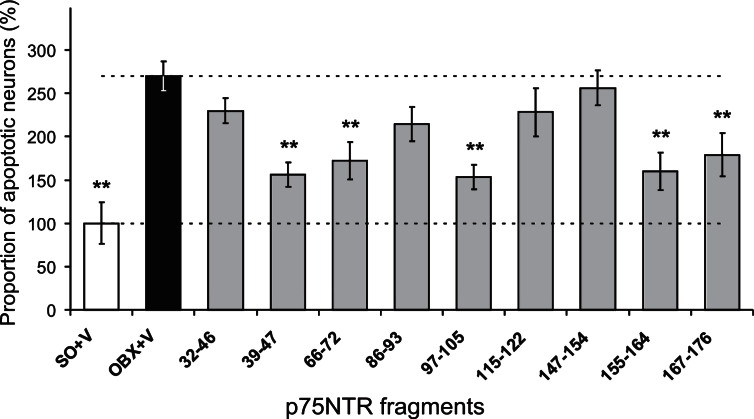
Increase of the apoptotic cell population in the *nucleus basalis magnocellularis* in OBX- versus SO-mice (OBX+V versus SO+V) and neuronal protective effect of the immunization against p75NTR fragments of 39–47, 66–72, 97–105, 155–164, and 167–176. Stars denote significant (*p* < 0.01, Mann-Whitney U-test) differences versus OBX. Error bars show 1 SEM.

In yhe NBM area, increased amount of apoptotic cells was found (Fig. 2) in brain slices from OBX- versus SO-mice (*n* = 136/25 versus 18/7,respectively) that was significant regardless whether the data from all OBX-mice or only from treated with p75NTR fragments were used (one-way ANOVA: F_10,143_ = 12.2 or F_9,132_ = 10.8, respectively, *p* < 0.001, for both). However, the immunized versus non-immunized OBX-mice demonstrated significant shrinking of apoptotic cell populations (one-way ANOVA: F9_,126_ = 7.9, *p* < 0.001) with evident beneficial effect for p75NTR fragments of 39–47, 66–72, 97–105, 155–164, and 167–176 (16/3, 16/3, 16/3, 12/3, and 12/3 slices/mice, respectively). In contrast to these, the treatments with 32–46, 86–93, 115–122, and 147–154 (16/4, 12/3, 8/2, and 16/3 slices/mice, respectively) were practically ineffective. Whether such immunization is beneficial for other brain structures, affected by bulbectomy, is analyzed in the next section.

### Morphological and functional characteristics of cortical and hippocampal neurons after immunization against different p75NTR fragments

**Fig.3:**
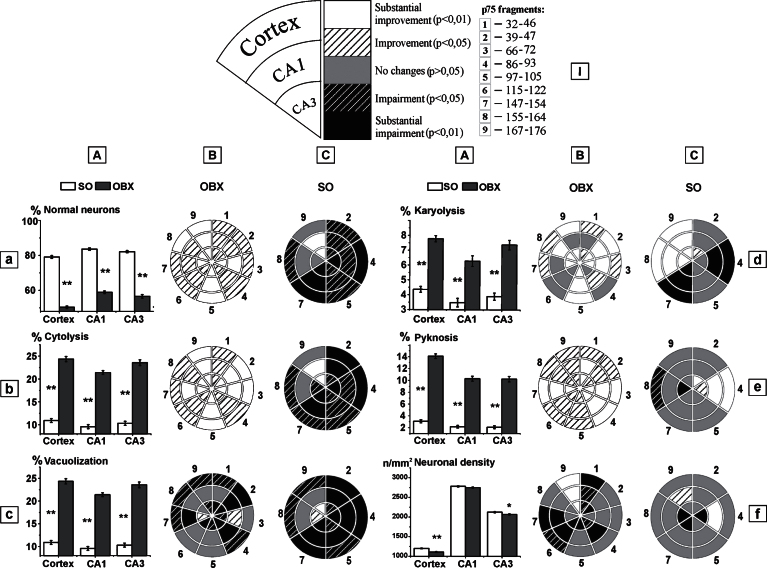
Morphological and functional characteristics of neurons in the temporal cortex and in the hippocampal areas of CA1 and CA3 after immunization against different p75NTR fragments. A) Statistical characterization of non-immunized SO-and OBX-mice, where stars denote significant (*p* < 0.01, Mann-Whitney U-test) difference between them. B) Radial presentation of the effects produced by the immunization against each of studied fragments of p75NTR in OBX-mice. C) The same in SO-mice. A description to the radial diagrams is shown on I. (Supporting experimental data in numerical format is presented in Supplementary Tables 1 and 2).

Recently, in OBX-mice, demonstrating typical AD hallmarks, pathological changes in the cortical and hippocampal neurons, closely associated with learning and memory processing, have been revealed [26]. The development of these cellular changes was characterized by nuclear fading (karyolysis) or shrinkage (pyknosis), membrane disintegration (cytolysis), intracellular vacuole formation (vacuolization), and by decreased neuronal density, resulting in neuronal death (Fig. 3A). Statistical analysis of these parameters in OBX-mice has revealed a predominantly protective influence of the immunization against the p75NTR fragments on the survival of cortical and hippocampal neurons (Fig. 3B). This immunological approach significantly improved their morphological and functional characteristics, i.e., increased the number of healthy neurons (Fig. 3B, a) at the expense of those with cytolysis, karyolysis, and pyknosis (Fig. 3B, b, d, and e, respectively). The beneficial effect of the immunization against p75NTR fragment of 167–176, expressed in the neuronal density (Fig. 3B, f), in particular, was evident in OBX- versus SO-mice (c.f., Fig. 3B, f versus Ñ, f, sector 9, both). In contrast, enlarged population of the vacuolated neurons (Fig. 3B, c) and decreased neuronal density in OBX-mice treated with 32–46, 115–122, and 147–154 p75NTR fragments (Fig. 3B, f, sectors 1, 6, and 7, respectively) was observed. Thus, protective effects of the immunization against p75NTR fragments in OBX-mice were expressed to a variable extent depending on the specificity of the fragments; however, a common non-specific tendency for some morphological characteristics was revealed as well. To define this more exactly, an additional series of experiments on 49 SO-mice was performed with immunization against most of the p75 NTR fragments used on OBX-mice: 39–47; 86–93; 97–105; 147–154; 155–164, and 167–176. With the exception of 167–176 p75NTR fragment (Fig. 3C, sector 9), the immunization against other fragments negatively affected the neuronal morphology in SO-mice, supporting the conclusion that this treatment specifically protects OBX-damaged brains from the pathology seemingly associated with enhanced Aβ level. Maximal negative changes in SO-mice were observed after the immunization against 86–93 and 147–154 p75NTR fragments (Fig. 3C, sectors 4 and 7, respectively) and were expressed in both decreased population of healthy neurons (Fig. 3C, a)and increased ones of pathological cells with visible cytolysis, vacuolization, and karyolysis (Fig. 3C, b-d). The changes in CA3 are especially impressive as they were observed at relatively low reference level of neuronal density in this area (Fig. 3A, f). The immunological treatment with 155–164 p75NTR fragment resulted in shrinkage of the karyolitic cell population both in the cortex and hippocampus (Fig. 3C, d, sector 8), whereas the populations of cells with other pathological features were increased exclusively in the cortex (Fig. 3C, b, c, e). Interestingly, the beneficial influence of the immunization against 167–176 p75NTR fragment on neuronal morphology in OBX-mice were observed, to a lesser extent, in SO-mice as well (c.f., Fig. 3B and C, a, b, d, sector 9). (For simplicity, the data associated with Fig. 3 has been included in the Supplementary materials.)

Whether the beneficial effects of the immunization on neuronal morphology is correlated with spatial memory improvement, as tested in Morris water maze, will be analyzed in the next section.

### Effects of the immunization against p75NTR fragments on spatial memory

**Fig.4:**
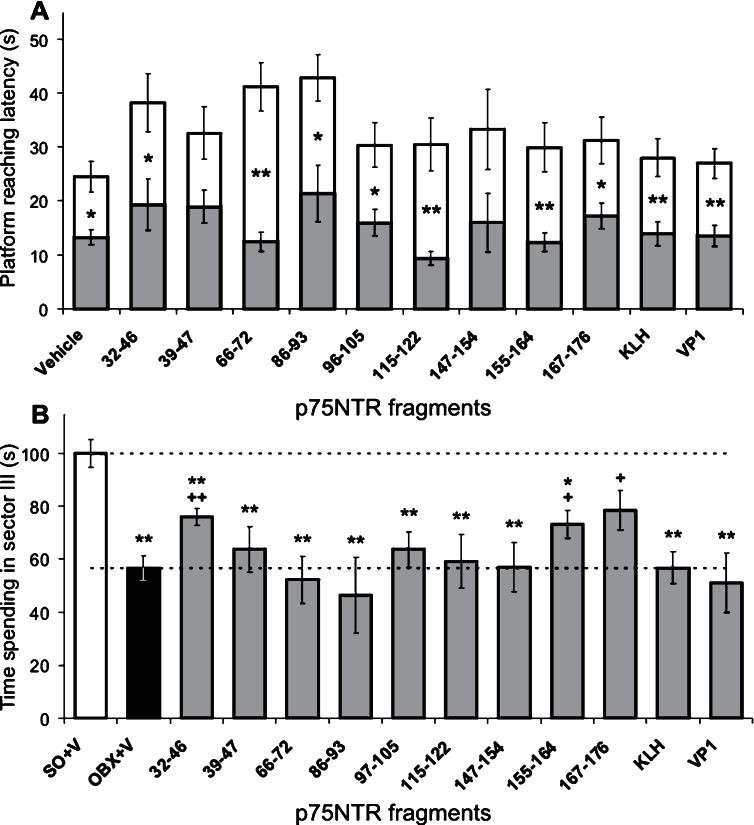
Learning (A) and memory (B) abilities of OBX-mice immunized against different fragments of p75NTR. A) Decreased latency in reaching the saving platform on the 4th day (gray bars) versus that on the 1st day (white bars) of learning is shown. Stars denote significant latency differences between the days: One and two markers are *p* < 0.05 and 0.01, respectively (Mann-Whitney U-test). B) In session with memory testing, relative (versus “SO+V” control) time spending in the sector where the saving platform was placed during learning was decreased in OBX-mice, with exception of partial recovery for those immunized against p75NTR fragments of 32–46, 155–164, and 167–176. Stars and pluses denote significant differences (one and two markers are *p* < 0.05 and 0.01, respectively, Mann-Whitney U-test) versus SO and OBX, respectively. A, B) V, KLH, and VP1 denote Vehicle, Keyhole Limpet Hemocyanin conjugate, and a protein from foot-and-mouth disease virus, respectively; error bars show 1 SEM.

**Fig.5:**
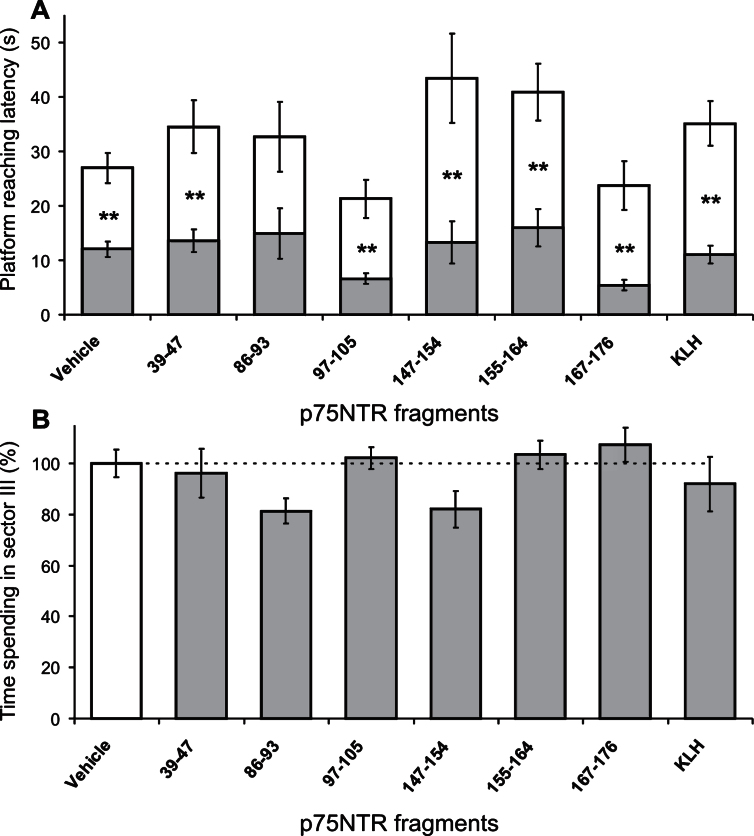
Learning (A) and memory (B) abilities of SO-mice immunized against different fragments of p75NTR. A) Decreased latency in reaching the saving platform on the 4th day (gray bars) versus that on the 1st day (white bars) of learning is shown. Stars denote significant (*p* < 0.01, Mann-Whitney U-test) latency differences between the days. B) In session with memory testing, relative (versus “Vehicle” control) time spending in the target sector was comparable in all groups of mice. A,B) KLH denotes Keyhole Limpet Hemocyanin conjugate; error bars show 1 SEM.

In the beginning of the main experiments, individual swimming capabilities of mice were evaluated in the water. Mean latencies to reach the visible platform for OBX- and SO-mice (*n* = 82 and 49, respectively) were approximately equal (7.3 ± 0.4 s and 8.1 ± 0.6 s, respectively; *t*-test, *p* > 0.05) regardless of whether they were immunized or not. In the following training days with the *hidden* platform, significant decrease of latencies in the 4th versus the 1st session was observed in both OBX- and SO-mice (Fig. 4A and 5A, two-way ANOVA: F_1,140_ = 104 and F_1,74_ = 71, respectively, *p*≤0.001, for both). Thus, the animals from each group have learned to reach the platform with reasonably latencies. OBX-mice were distributed within the groups as follows (the number of mice): Without (16) and after the immunization with synthetic peptides, the analogs of the p75NTR fragments of 32–46 (5), 39–47 (6), 66–72 (7), 86–93 (6), 97–105 (7), 115–122 (6), 147–154 (4), 155–164 (7), 167–176 (7), KLH (6), and VP1 (5). SO-mice did the same as follows: Without the immunization (15) and after the immunization with the p75NTR fragments of 39–47 (5), 86–93 (3), 97–105 (5), 147–154 (3), 155–164 (4), 167–176 (5), KLH (5), and VP1 (4).

The memory testing revealed that the trained OBX-mice from all groups spent significantly less time in the target sector III than SO-mice (one-way ANOVA: F_12,83_ = 8.9, *p* < 0.001), however, OBX-mice immunized against 32–46, 155–164, and 167–176 p75NTR fragments demonstrated partial memory improvement versus non-immunized OBX-animals (Fig. 4B). The immunization against these fragments has been shown above to be responsible for the beneficial effect on the functioning of acetylcholine-synthesized neurons in the basal forebrain (see Fig. 1A). Furthermore, the treatment with 155–164 and 167–176 p75NTR fragments evidently rescued morphology of cortical and hippocampal neurons (see Fig. 3, a, b, d, e, sectors 8 and 9). In contrast, spatial memory in SO-mice was insensitive (one-way ANOVA: F_7,37_ = 1.9, *p* > 0.05) to the immunization against those fragments which were effective for OBX-mice (c.f., Figs. 4B and 5B). Moreover, the treatment with 86–93 and 147–154 p75NTR fragments tended to impair the memory in SO-mice, and this was well correlated with the negative effects on neuronal morphology in the cortex and hippocampus (see Fig. 3C, a - d, sectors 4 and 7).

Therefore, it is reasonable to conclude that, in OBX- versus SO-mice, identical p75NTR fragments are differently involved in different ways in memory functioning and neuronal organization in the brain. The only exception is associated with 167–176 fragment whose immunological blockade produced the cholinergic and morphological protective effects both in OBX- and, partially, in SO-mice.

## DISCUSSION

In this study, the effects of the immunization against several selected p75NTR fragments in OBX-mice, characterized by typical hallmarks of Alzheimer-type neurodegeneration, were analyzed to reveal the most effective path to develop a new perspective approach for AD treatment. We suggest that the observed effects of the immunization were predominantly initiated by a blockade of the fragment-associated binding sites in the extracellular domain of p75NTR by appropriate antibodies whose titers were sufficiently high in our study (Table 1).

The immunization against some of p75NTR fragments in OBX-mice improved their spatial memory, lowered cholinergic deficit and number of neurons with apoptosis in the basal forebrain, and normalized cellular morphology in the cortex and hippocampus. Maximal positive effects were observed after the immunization against 155–164 and 167–176 amino acid residues of p75NTR. One negative consequence of this treatment became apparent in the decrease of neuronal density, especially, in the hippocampus (Fig. 3B, f) that seemingly was associated with disturbances in the apoptotic or, alternatively, autophagy mechanism, noticed at the AD progression [30]. The increased density of ChAT-immunopositive neurons in the forebrain observed in our study after immunization against some p75NTR fragments (Fig. 1A) is in line with evidence both of enhanced cholinergic innervations in the cortex and hippocampus in knock-out-mice (ChAT-cre p75(in/in) lacking p75NTR [31] and increased numbers of such neurons [32]. In general, our results are in line with evidence of the critical role of p75NTR in the degeneration of cholinergic neurons evoked by exposure either to Aβ [13, 18], or to pro-/neurotrophins [33]. Indeed, the antibodies against the *extracellular* p75NTR have been shown to inhibit Aβ-induced cell death and significantly diminished cell loss [20]. Furthermore, the immunization against some p75NTR fragments in OBX-mice was accompanied in our study by diminished numbers of apoptotic neurons in the cholinergic areas of their forebrains (Fig. 2). Moreover, enhanced expression of both p75NTR, its major ligand, pro-neurotrophin, and Aβ, in AD brains [15, 34], suggest that antibodies against binding sites for the ligands, inducing apoptotic processes, will be able to improve memory in OBX-mice and neuronal morphology in their brains.

We have revealed that the most protective effect in OBX-mice was produced by the immunological blockade of 155–164 and 167–176 fragments of p75NTR. However, in spite of practically identical roles of these amino acid residues for survival/death of ChAT-containing neurons (Fig. 1A), the protective effects of their blockade may originate from different sources. We have shown recently that the immunological blockade of 155–164 residues in p75NTR of OBX-mice, characterized by enhanced level of Aβ [29], was accompanied by significant decrease of the brain Aβ level, whereas immunization against the 167–176 residue was ineffective [35]. Thus, the 155–164 fragment of the p75NTR extracellular domain may specifically be involved in the mechanisms of either Aβ binding with p75NTR or Aβ aggregation [36], or, alternatively, in guiding Aβ to lysosomes for consecutive degradation in cholinergic neurons [11]. Regardless of the mechanisms associated with the lowering of Aβ in the brain, the immunological blockade of the 155–164 residue may be useful approach for the brain protection from AD-linked cholinergic deficit and memory impairment, in particular, by the improving of the memory consolidation provided by synapse remodeling [37].

In summary, in spite of some differences in the effects of the immunization against various p75NTR fragments in OBX-mice, evident similarities were observed in morphological characteristics of cortical and hippocampal neurons (Fig. 3B), that might be considered as a non-specific action of the active immunization. However, this approach applied to SO-mice showed either no or opposite effects. This suggests that the pathological/neurodegenerative processes in OBX-mice are accompanied by changes in the role of the studied fragments of p75NTR extracellular domain in cell survival. Thus, the immunological blockade of p75NTR 155–164 fragment exerts either a negligible or negative influence on neuronal characteristics in SO-mice, whereas improvements of both memory and neuronal morphology, including increased number of acetylcholine-producing cells, in the forebrain were evident. In contrast, the treatment with 86–93 and 147–154 p75NTR fragments impaired the memory and neuronal characteristics in SO-mice without significant negative influences on OBX-mice. Interestingly, the immunization against p75NTR 167–176 fragment was accompanied by positive effects in both OBX- and SO-mice. The structural basis for these may include various conformational transformations after the blockade of neurotrophin or proneurotrophin binding to p75NTR. In this case, conformational mechanisms apparently have to be sensitive to the extracellular domain structures of the receptor. It is well known that dimeric p75NTR is able to bind to NT-4 receptor with low affinity and to stimulate neurogenesis. Moreover, p75NTR, in conjunction with dimeric TrkA, TrkB, and TrkÑ receptors, has shown to enhance efficacy of their binding with neurotrophic factors such as NGF, BDNF, NT-3, and NT-4 [38] to enhance, in turn, the survival rate of cells.

In conclusion, a study of interactive functioning of p75NTR and Trk receptors by using of immunological means may be a key approach for both the understanding of mechanisms of neuronal degeneration and the developing of effective new treatment applicable for AD [13], aging [39] and other neurodegenerative diseases associated with cholinergic system malfunctioning in the brain.

## CONCLUSION

In olfactory bulbectomized rats with typical hallmarks of Alzheimer-type of degeneration, we have demonstrated protective effects of immunization against two of nine specific fragments of p75 neurotrophin receptor extracellular domain on both cholinergic neurons in the forebrain and cognitive abilities in the animals.

## Supplementary Material

Supplementary Material

## Figures and Tables

**Table 1:** Serum antibody titers in the olfactory-bulbectomized mice immunized against different synthetic p75 fragments conjugated with the KLH-peptide

	Serum antibody titers (in -lg of sera dilution) versus p75NTR fragments								
P75NTR fragment	*32*–*46*	*39*–*47*	*66*–*72*	*86*–*93*	*97*–*105*	*115*–*122*	*147*–*154*	*155*–*164*	*167*–*176*
Antibody titer	3.8–4.1	3.4–4.1	3.1–3.4	3.1–3.4	3.7–4.1	2.2–2.8	2.2–2.5	3.1–3.8	3.4–4.1

## Appendix

The supplementary material is available in the electronic version of this article: http://dx.doi.org/10.3233/JAD-160146.
