# Supplementary material for: Immunization Against Specific Fragments of Neurotrophin p75 Receptor Protects Forebrain Cholinergic Neurons in the Olfactory Bulbectomized Mice
Source: J Alzheimers Dis. 2016 Jun 22;53(1):289–301. doi: 10.3233/JAD-160146 (PMC4942728; doi:10.3233/JAD-160146)
Supplement: Supplementary Material [file jad-53-jad160146-s001.docx]

**Supplementary Table 1.** Morphological and functional characteristics of neurons in the temporal cortex and in the hippocampal areas of CA1-CA2 and CA3-CA4 in olfactory-bulbectomized (OBX) mice after immunization against different p75NTR fragments.

|  | Group  Of mice | Neuronal density (n/mm^2^) | Normal neurons  (%) | Pathological neurons (%) | | | |
| --- | --- | --- | --- | --- | --- | --- | --- |
|  |  |  |  | Pyknosis | Cytolysis | Karyolysis | Vacuolization |
| Temporal cortex | SO  OBX  (32-46)  (39-47)  (66-72)  (86-93)  (97-105)  (115-122)  (147-154)  (155-164)  (167-176) | 1200±15.7**  1110±16.1  883±12.8**  1122±7.9  1099±8.3  971±12.9**  1123±8.7  1049±18.4*  950±24.4**  1113±8.9  1298±9.1** | 79.1±0.80**  50.4±0.73  63.6±0.39  69.6±0.96**  73.5±0.40**  65.9±0.44**  75.0±0.62**  67.7±0.89**  71.6±1.05**  69.9±0.69**  74.0±1.38** | 3.1±0.26**  14.1±0.34  4.4±0.25**  4.1±0.23**  2.4±0.18**  2.4±0.21**  3.8±0.34**  3.8±0.31**  2.4±0.49**  5.6±0.31**  4.2±0.26** | 10.9±0.43**  24.3±0.57  20.9±0.25**  13.7±0.49**  15.3±0.44**  19.9±0.27**  13.1±0.33**  18.3±0.47**  18.4±0.58**  14.6±0.39**  11.7±0.63** | 4.4±0.21**  7.3±0.21  5.4±0.23**  4.4±0.23**  5.1±0.19**  6.5±0.19  4.1±0.21**  6.1±0.34*  4.8±0.42**  5.5±0.25**  4.5±0.27** | 1.7±0.15**  3.9±0.3  5.7±0.23**  8.1±0.59**  3.7±0.21  5.2±0.23**  3.9±0.29  4.2±0.29  2.8±0.33*  4.7±0.22*  5.6±0.61* |
| СА1-СА2 (Hippocampus) | SO  OBX  (32-46)  (39-47)  (66-72)  (86-93)  (97-105)  (115-122)  (147-154)  (155-164)  (167-176) | 2782±21.1*  2749±22.3  2693±13.9  2741±18.4  2783±14.3  2664±13.3**  2781±11.7  2652±17.5*  2656±32.4*  2745±14.6  2814±18.8* | 83.6±0.68**  58.9±0.79  73.2±0.47**  74.4±0.70**  80.5±0.46**  69.8±0.46**  80.9±0.91**  70.2±0.55**  75.8±1.08**  75.8±1.02**  73.9±0.65** | 2.2±0.22**  10.3±0.41  1.3±0.13**  2.6±0.25**  1.0±0.13**  1.1±0.15**  2.4±0.31**  2.1±0.31**  0.9±0.35**  3.6±0.27**  3.6±0.24** | 9.6±0.43**  21.4±0.42  16.2±0.31**  13.7±0.36**  12.4±0.28**  19.3±0.27**  10.7±0.43**  17.6±0.27**  11.6±0.81**  13.0±0.51**  12.8±0.32** | 3.5±0.29**  6.3±0.37  5.8±0.33  3.2±0.21**  3.8±0.21**  4.4±0.22**  3.2±0.25*  6.3±0.26  6.3±0.37  4.6±0.22**  5.6±0.27 | 1.2±0.17**  3.1±0.24  3.6±0.19  6.1±0.52**  2.2±0.21*  5.8±0.21**  2.8±0.27  3.7±0.24  5.4±0.56*  3.4±0.41  4.0±0.45 |
| СА3-СА4 (Hippocampus) | SO  OBX  (32-46)  (39-47)  (66-72)  (86-93)  (97-105)  (115-122)  (147-154)  (155-164)  (167-176) | 2123±20.5  2066 ± 21.1  2037±11.2  2008±14.1*  2045±9.7  2089±11.3  2006±11.2*  2011±12.9*  2042±34.5  2053±14.2  2119±10.3* | 82.1±0.63**  56.5±1.00  68.5±0.82**  75.4±0.73**  79.2±0.53**  66.6±0.37**  78.1±0.74**  69.1±1.80**  77.6±0.92**  78.1±1.01**  77.3±1.26** | 2.1±0.23**  10.2±0.46  1.4±0.16**  3.9±0.37**  1.1±0.13**  0.9±0.12**  2.9±0.26**  1.5±0.19**  1.3±0.37**  3.4±0.24**  2.8±0.23** | 10.3±0.44**  23.5±0.62  18.3±0.45**  11.1±0.42**  13.0±0.30**  20.4±0.25**  11.9±0.44**  16.1±0.43**  12.2±0.51**  12.4±0.58**  10.8±0.70** | 3.9±0.24**  7.4±0.32  6.1±0.27*  3.5±0.24**  3.3±0.19**  6.6±0.22  3.5±0.21**  5.9±0.22**  4.4±0.37**  3.4±0.31**  4.3±0.27** | 1.6±0.19*  2.3±0.21  5.9±0.28**  6.0±0.32**  3.4±0.21**  5.6±0.21**  3.4±0.21*  6.0±0.25**  4.5±0.43**  2.8±0.23  4.8±0.43** |

Notes: Statistical characterization (mean ± SEM) of OBX-mice immunized against p75NTR fragments (shown in brackets), where stars denote significant (*p < 0.05; ** р < 0.01, Mann-Whitney U-test) difference versus "OBX" (underlined) group.

**Supplementary Table 2.** Morphological and functional characteristics of neurons in the temporal cortex and in the hippocampal areas of CA1-CA2 and CA3-CA4 in sham-operated (SO) mice after immunization against different p75NTR fragments.

|  | Group  of mice | Neuronal density (n/mm^2^) | Normal neurons  (%) | Pathological neurons (%) | | | |
| --- | --- | --- | --- | --- | --- | --- | --- |
|  |  |  |  | Pyknosis | Cytolysis | Karyolysis | Vacuolization |
| Temporal cortex | Vehicle  (39-47)  (86-93)  (97-105)  (147-154)  (155-164)  (167-176) | 1200±15.7  1193±12.8  1181±13.7  1179±10.9  1175±12.8  1181±13.9  1198±15.0 | 79.1±0.80  74.2±0.48**  68.8±0.52**  73.5±0.40**  71.1±0.33**  73.9±0.47**  79.2±0.86 | 3.1±0.26  2.8±0.25  1.0±0.19**  2.7±0.25  2.7±0.18  4.2±0.24*  3.8±0.28 | 10.9±0.44  13.8±0.28**  15.9±0.36**  13.4±0.31**  14.3±0.22**  14.3±0.25**  10.3±0.46 | 4.4±0.21  4.5±0.2  8.1±0.28**  5.0±0.19  6.2±0.22**  3.5±0.20*  3.5±0.20* | 1.7±0.2  4.6±0.27**  6.3±0.25**  5.5±0.23**  5.6±0.25**  4.0±0.24**  3.3±0.27** |
| СА1-СА2 (Hipp) | Vehicle  (39-47)  (86-93)  (97-105)  (147-154)  (155-164)  (167-176) | 2782±21.1  2737±14.4  2843±20.2*  2828±16.3  2762±11.7  2753±15.3  2819±18.8 | 83.6±0.68  75.5±0.69**  74.1±0.87**  75.0±0.60**  69.3±0.40**  84.1±0.44  89.5±0.49** | 2.2±0.25  2.6±0.29  1.0±0.22**  1.7±0.17  1.7±0.25  2.3±0.21  1.9±0.21 | 9.6±0.43  13.9±0.39**  14.7±0.54**  13.9±0.36**  16.0±0.34**  10.5±0.32  4.6±0.20** | 3.5±0.29  2.6±0.29  5.8±0.45**  4.1±0.28  8.0±0.24**  2.1±0.21*  2.4±0.22* | 1.2±0.17  4.7±0.23**  4.4±0.25**  5.4±0.21**  5.0±0.28**  1.8±0.19  1.7±0.18 |
| СА3-СА4 (Hipp) | Vehicle  (39-47)  (86-93)  (97-105)  (147-154)  (155-164)  (167-176) | 2123±20.45  2118 ± 8.6  2028±17.9**  2091±12.9  2002±11.6**  2042±14.5*  2131±13.0 | 82.1±0.63  76.5±0.39**  67.2±0.58**  77.4±0.50**  66.7±0.51**  82.5±0.40  90.7±0.43** | 2.1±0.24  3.1±0.24*  1.1±0.24*  1.6±0.17  2.2±0.21  2.8±0.20  1.9±0.17 | 10.3±0.44  13.9±0.29**  16.7±0.31**  13.0±0.28**  16.8±0.3**  11.9±0.3*  4.4±0.28** | 3.9±0.40  2.7±0.29*  5.3±0.98  4.0±0.19  9.4±0.22**  2.1±0.35**  2.1±0.20** | 1.6±0.19  3.6±0.19**  5.6±0.32**  3.9±0.23**  4.8±0.33**  2.5±0.22*  1.2±0.15 |

Notes: Statistical characterization (mean ± SEM) of SO-mice immunized against p75NTR fragments (shown in brackets), where stars denote significant (*p < 0.05; **р < 0.01, Mann-Whitney U-test) difference versus the "Vehicle" (underlined) group. The hippocampus is abbreviated as Hipp.
